# Supplementary figures and images for: Electrical Stimulation of Adipose-Derived Stem Cells in 3D Nanofibrillar Cellulose Increases Their Osteogenic Potential
Source: Biomolecules. 2020 Dec 18;10(12):1696. doi: 10.3390/biom10121696 (PMC7766661; doi:10.3390/biom10121696)

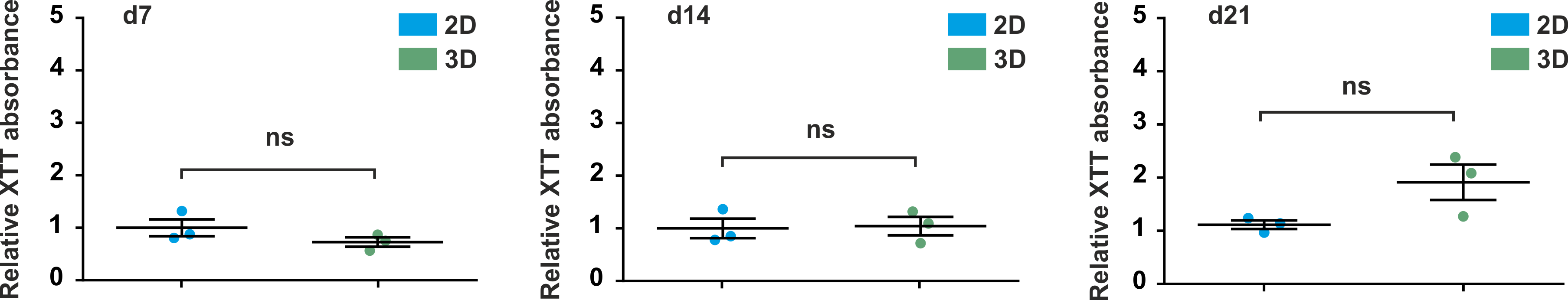

Supplement: Supplementary file 1 [file biomolecules-10-01696-s001.zip › biomolecules-1041454-supplementary.tif]
